# Shielding effectiveness of multilayer laminate of aluminum metal matrix and micro absorbing materials

**DOI:** 10.1016/j.mex.2023.102172

**Published:** 2023-04-06

**Authors:** Srinu Budumuru, Sankranti Srinivasa Rao, Durgarao Jenjeti, T Venkata suri apparao

**Affiliations:** Department of electrical electronics and communication engineering, GITAM University, Visakhapatnam, India

**Keywords:** Transmission/Reflection method, Shielding, Laminate, Absorbing material, Hardness, Tensile strength

## Abstract

Shielding blocks are widely used to protect sensitive electronic components in defense and medical equipment. In many circumstances, acquiring shielding effectiveness is insufficient, with shielding effectiveness having higher priority if the material possesses mechanical characteristics. To achieve the required electromagnetic shielding effectiveness and mechanical properties such as tensile strength and hardness, a laminate sheet with Al6061 material and micro absorbing materials such as MNZC ferrite rubber composite, Ca-NiTi hexaferrite composites, and Carbonyl iron is preferred in the current work. The total work organized as

• Selection of the aluminum metal matrix and micro absorbing material.
• Finding the electrical parameters using the vector network analyzer (VNA) using Transmission/Reflection method.
• The shielding performance was evaluated for different laminate thicknesses at the X-band.
• The material mentioned above can be used as a shielding building block in aeronautical applications and near sensitive medical equipment.

## Graphical abstract

**Figure:**
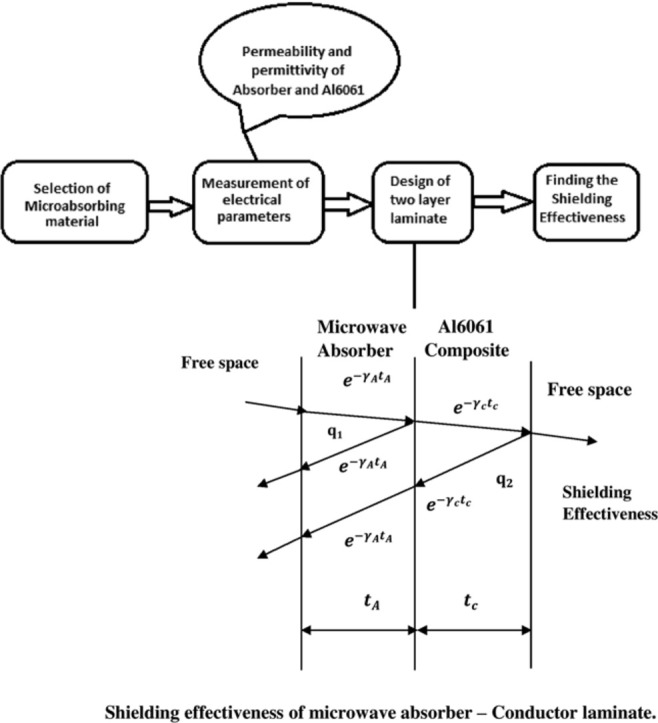

## Related research article

High intensity radiated field protection layer design with Al6061 metal matrix material reinforced with SiC and fly ash, Advances in Materials and Processing Technologies [1]. DOI: 10.1080/2374068X.2022.2115441

Specifications table

**Table:** 

Subject area:	Engineering
More specific subject area:	EMI/EMC of Materials
Name of your method:	Transmission/Reflection method
Name and reference of original method:	High-intensity radiated field protection layer design with Al6061 metal matrix material reinforced with SiC and fly ash, Advances in Materials and Processing Technologies, DOI: 10.1080/2374068X.2022.2115441.
Resource availability:	[1]. DOI: https://doi.org/10.1080/2374068X.2022.2115441

## Method details

### Overview

As electronics and wireless technology advance, electromagnetic radiation becomes a serious problem that affects the functioning of electronic devices and may harm humans and animals. The only solution for protecting electronic and sensitive devices from electromagnetic devices is providing shielding blocks around those. Shields that are light in weight and physically strong are generally preferred for most aerospace, military, and commercial applications, with current researchers focusing on them. Metals generally have the finest electromagnetic shielding qualities; however, due to their weight, they are rarely taken into consideration in many applications. At lower frequencies, aluminum metals function well as shields, and the shielding characteristics fluctuate depending on the structure [2,3]. Foam-type structures can be used as an alternative to direct metals for effective shielding, but they lack the necessary physical strength for many applications. While Zhengbin Xu et al. worked with aluminum foams of various porosities to achieve good shielding capabilities, the materials' strength was insufficient for aerospace industry use. They only operated at extremely low frequencies [4]. Due to their flexibility when arranging around the equipment and lightweight, superior corrosion resistance, and magnetic and mechanical qualities, polymeric materials are frequently utilized as electromagnetic shielding materials [5]. Wang. et al. reviewed polymeric materials and discovered various shielding values for multiple frequencies. However, the electric conductivity of polymeric materials is inferior, resulting in poor transmission of electric flux through it [6,7].

Composite materials are essential in today's technology because they provide superior shielding while still having high mechanical characteristics. MMCs (Metal Matrix Composites) are materials that have been reinforced with other metals, organic compounds, and ceramic elements. Supporting details increase pure metal's shielding properties and strength [8,9]. The material with strong conductivity gives superior shielding in terms of reflection, whereas the material with good permeability provides good absorption qualities and potential for shielding enhancement [10]. The prospect of micro absorbers is improved by balancing dielectric and magnetic losses of material by reinforcing magnetic and dielectric material into alloys [11]. The majority of conductors have strong conductivity and provide decent shielding; however, they are prone to corrosion. Because of its electrical characteristics, fluidity, castability, corrosion resistance, and high strength-weight ratio, the Al6061 composite was employed in this study [12,13]. A micro absorbent material supported by a conductor laminate provides superior shielding to standard metal-based shielding [14]. Tahar Merizgui**,** Bachir Gaoui et al. worked on multilayer laminate and employed thickness enhancement to improve shielding efficiency as the number of layers increased [15,16]. There is a large requirement of shielding material with low weight and high strength in terms of mechanical properties to protect the sensitive electronic equipment's and communication devices. For that in the present work introduced a conductor supported by a two-layer laminate containing various micro-absorbing materials [17]. Micro-absorbing materials allow electromagnetic waves to pass readily from free space into them while attenuating the signal within. The microwave signal is absorbed as a result of attenuation. To achieve the needed shielding, three varieties of micro absorbing material were employed with the help of Al6061. MNZC ferrite rubber composite, Ca-NiTi hexaferrite composites, and carbonyl iron were used as absorbent materials. These materials have strong shielding qualities while being very light [18].

Al6061 metal matrix composites are excellent shielding materials with excellent mechanical and electrical characteristics [19,20].

• Using a Vector Network Analyzer (VNA) based on Transmission/Reflection method, the electrical properties of the absorbing material's permeability and permittivity were examined.
• For the protection of sensitive electronic equipment, a two-layer laminate of micro-absorbing material supported by Al6061 composite material was designed.
• The permeability and permittivity data were used to calculate the laminate's shielding effectiveness.

### Procedure of transmission/reflection method for measuring the electrical parameters of material

**Fig. 1:**
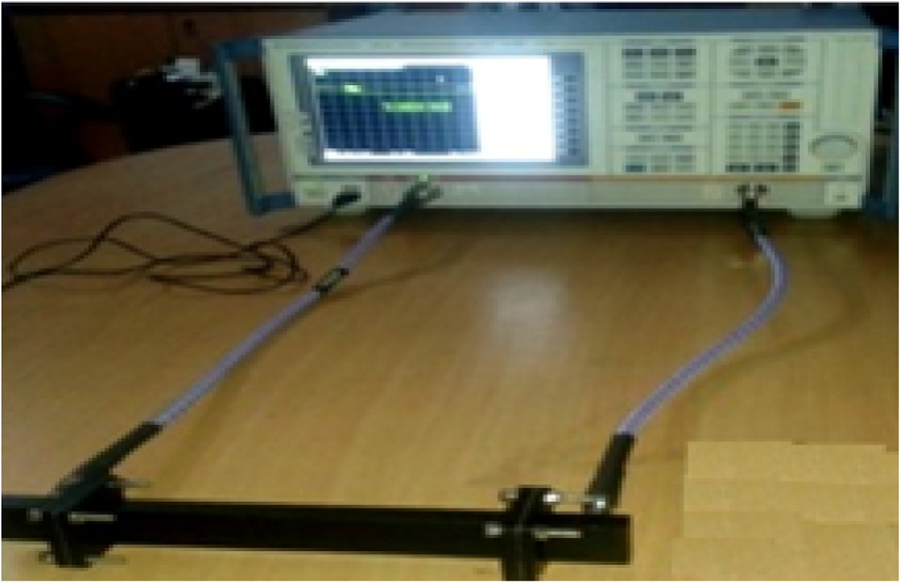
Experimentation setup of transmission/reflection method.

Fig. 1 represents the vector network analyzer setup to measure the electrical parameters using Transmission/Reflection method.

• The material for which need to find the electrical parameters can placed in the wave guide connected to the VNA.
• Based on the transmission and reflection of the electromagnetic waves the electrical parameters of both micro absorbing materials and Al6061 material was measured.

### Procedure for measuring shielding effectiveness

• Measure the intrinsic impedance of each layer.
• Measure the transmission coefficient of each boundary.
• Measure reflection coefficient of each boundary.
• Measure impedance right to each interface
• Measure total transmission coefficient.
• Measure total shielding effectiveness.

**Fig. 2:**
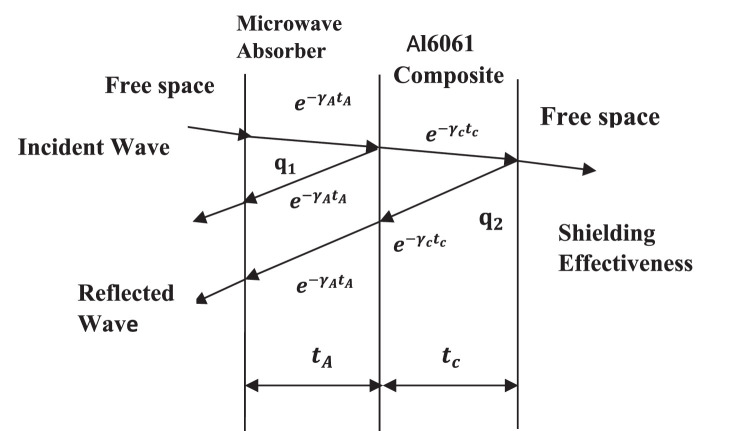
Shielding effectiveness of microwave absorber –conductor laminate.

Fig. 2 depicts the shielding effectiveness of a two-layer laminate of micro absorbing material supported by Al6061 composite material. The electromagnetic wave strikes the laminate from free space and enters the microwave absorbing material, where it is significantly attenuated [21]. Following an incident on an Al6061 composite, some energy enters the free room by penetrating the Al6061 material, while the residue can be reflected. The microwave absorbing material absorbs the reflected signal once again [22]. Shielding effectiveness is a combination of absorption and reflection of electromagnetic wave through the composite material [23,24].

The transmission coefficient of absorber conductor and conductor free space boundaries are given as [25]

(1) $$p=\frac{16\eta_{O}\eta_{A}\eta_{M}}{\left(\eta_{0}+\eta_{A}\right)\left(\eta_{A}+\eta_{M}\right)\left(\eta_{M}+\eta_{0}\right)}$$

The intrinsic impedance of free space $\eta_{0}$ is 120π, and the intrinsic impedance of metal matrix conductor $\eta_{M}\;$can be estimated as

(2) $$\eta_{M}=\left(1+j\right)\sqrt{\frac{\pi f\mu }{\sigma }}$$

Whereσandμ are the conductivity and permeability of the Al6061.

Reflection coefficient at absorbing material-Metal matrix conductor interface is$\;q_{1}$ and metal matrix conductor - free space interface is $q_{2}$ can be given as [26]

(3) $$q_{1}=\frac{\left(\eta_{A}-\eta_{0}\right)\left(\eta_{A}-z\left(M\right)\right)}{\left(\eta_{A}+\eta_{O}\right)\left(\eta_{A}+z\left(M\right)\right)}\;\;q_{2}=\frac{\left(\eta_{M}-\eta_{A}\right)\left(\eta_{M}-\eta_{0}\right)}{\left(\eta_{M}+\eta_{A}\right)\left(\eta_{M}+\eta_{0}\right)}$$

wherez(M) are the impedance to the right of the absorber-metal matrix conductor interface, respectively., $z\left(M\right)=\eta_{M}\left[\frac{\eta_{0}cosh\left(\gamma_{M}t_{M}\right)+\eta_{M}sinh\left(\gamma_{M}t_{M}\right)}{\eta_{M}cosh\left(\gamma_{M}t_{M}\right)+\eta_{0}sinh\left(\gamma_{M}t_{M}\right)}\right]$ (4) where $\;t_{M}$ are the thicknesses of the metal matrix conductor and $\;\gamma_{M}\;$are the propagation constants metal matrix conductor [27], respectively

(5) $$\gamma_{M}=\left(1+j\right)\sqrt{\left(\pi f\mu_{M}\sigma_{M}\right)}$$

Considering successive re-reflections at the interface of the two layers, the total transmission coefficient across the laminate can thus be derived to be

$T=p\;{\left[\left(1-q_{1}e^{-2\gamma_{A}t_{A}}\right)\left(1-q_{2}e^{-\gamma_{M}t_{M}}\right)\right]}^{-1}e^{-\gamma_{A}t_{A}-\gamma_{M}t_{M}}\;\left(6\right)\;$The Shielding effectiveness of the three-layer laminate can thus be expressed in decibels as

(7) $$S=-20log_{10}\left(T\right)\;dB\;$$

## Results of vector network analyzer (VNA)

**Fig. 3:**
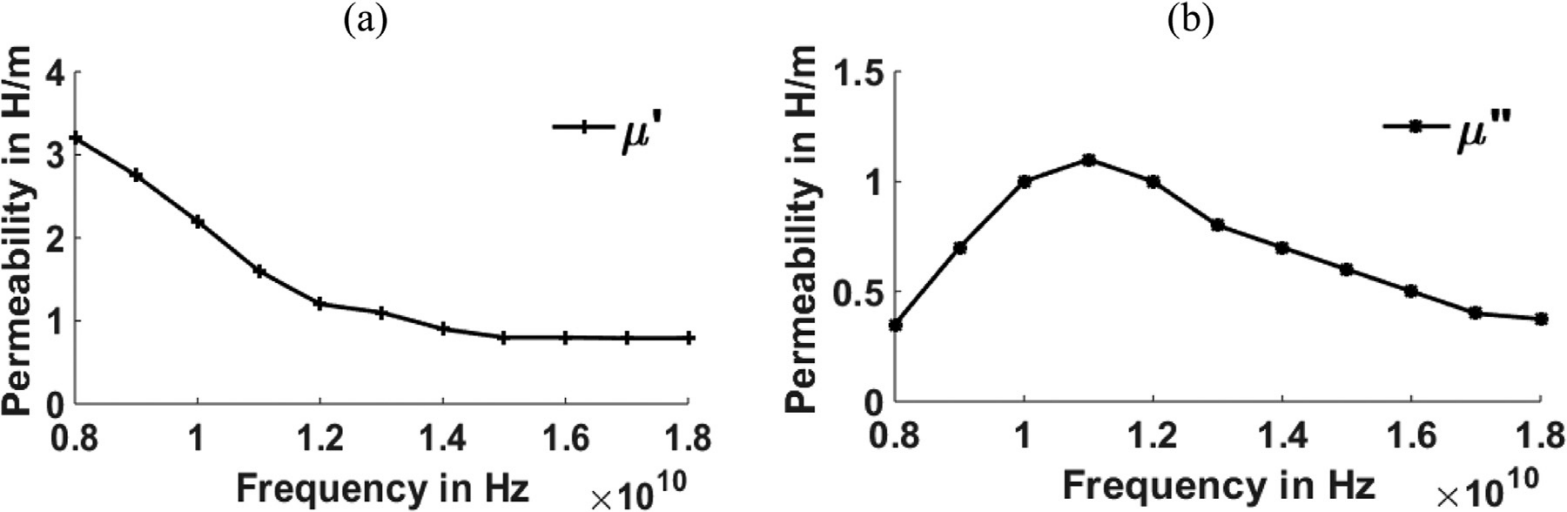
(a), (b) Relation between Permeability and frequency for MNZC ferrite rubber composite.

**Fig. 4:**
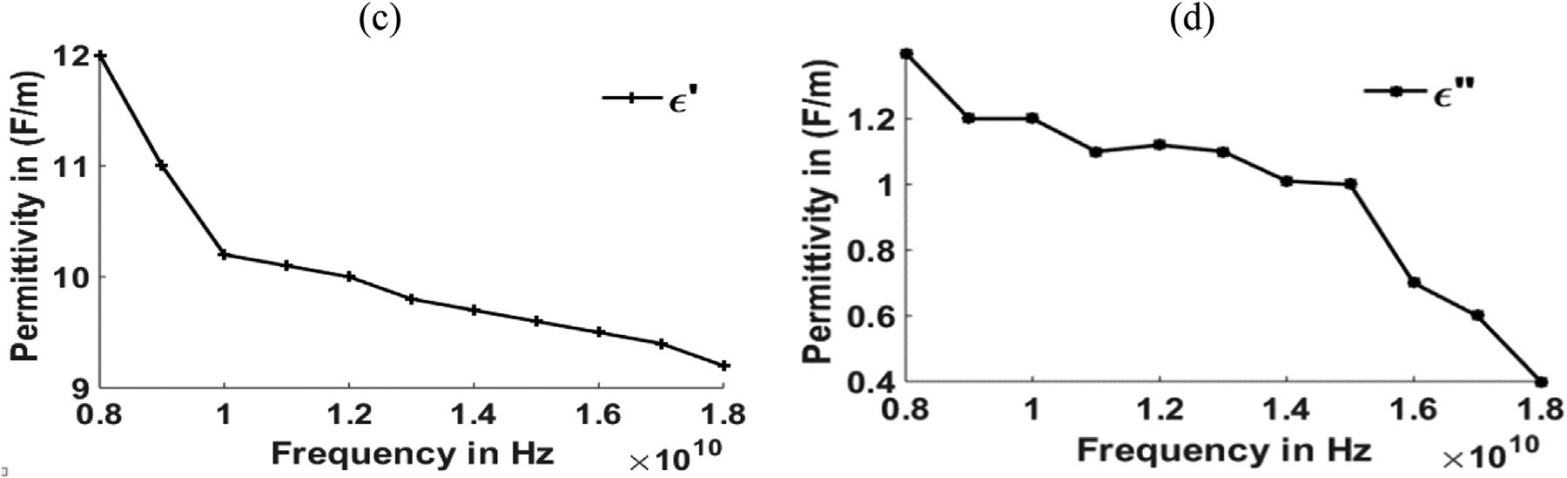
(c), (d) Relation between Permittivity and frequency for MNZC ferrite rubber composite.

**Fig. 5:**
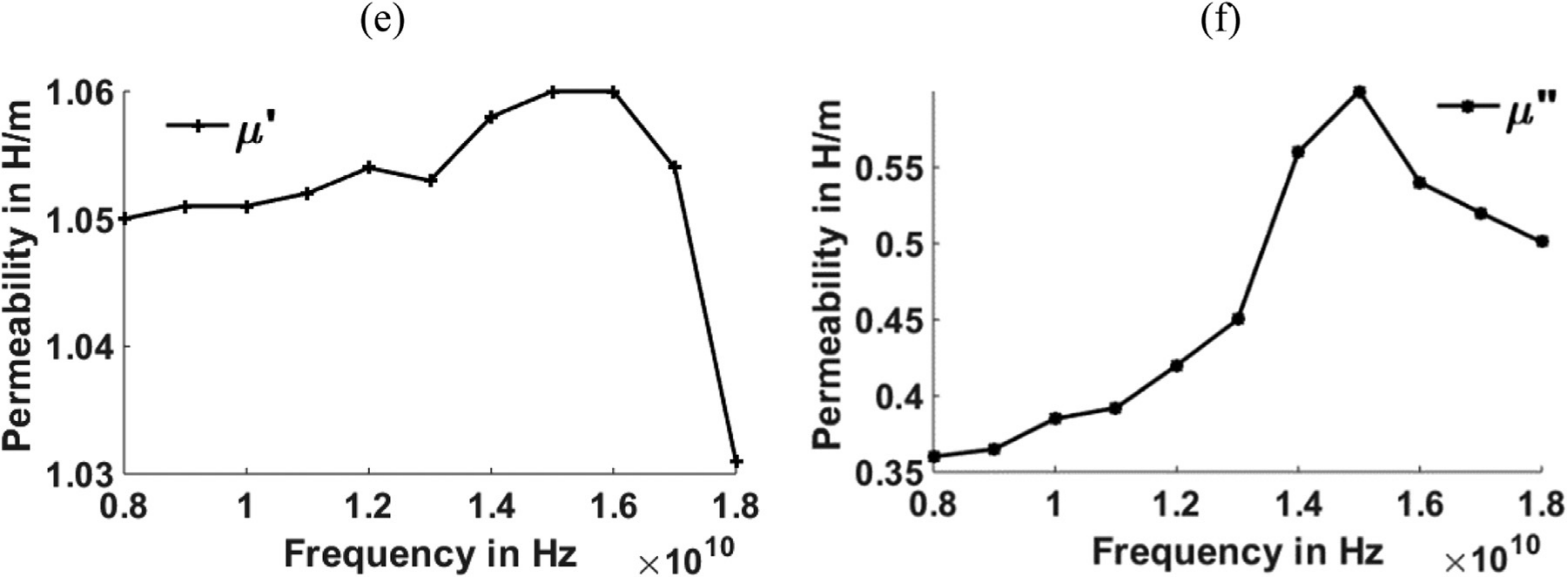
(e), (f) Relation between Permeability to frequency for Ca–NiTi hexaferrite composites.

**Fig. 6:**
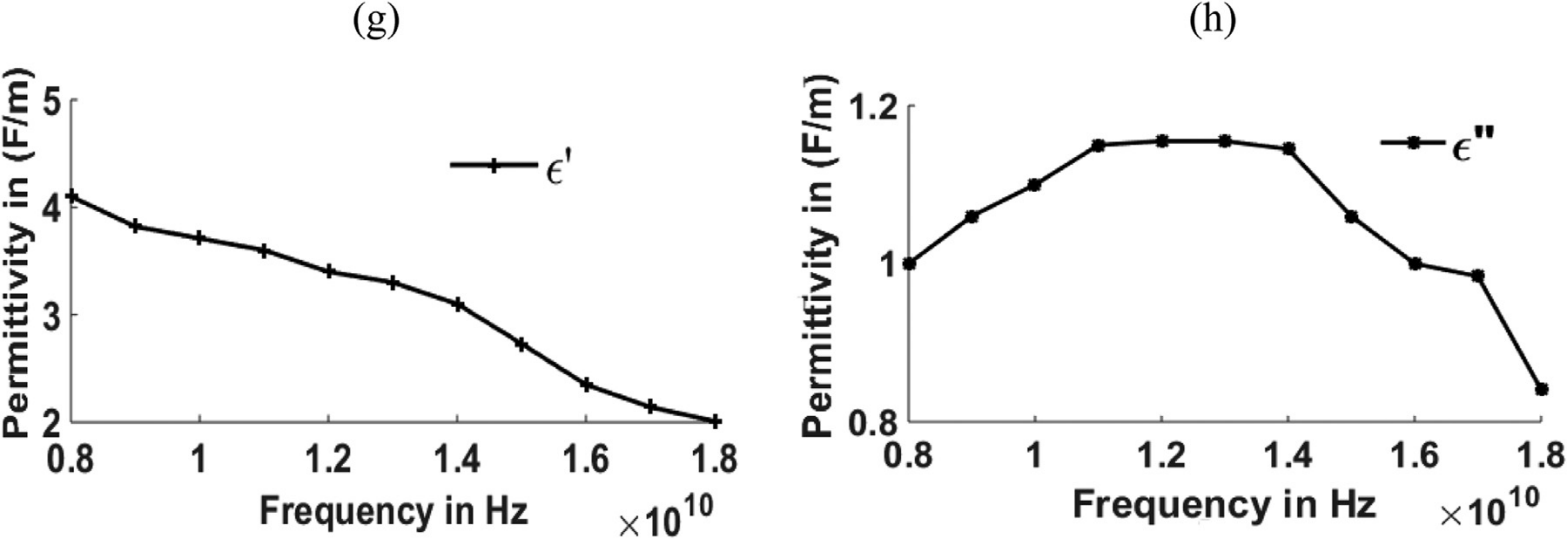
(g), (h) Relation between Permittivity to frequency for Ca–NiTi hexaferrite composites.

**Fig. 7:**
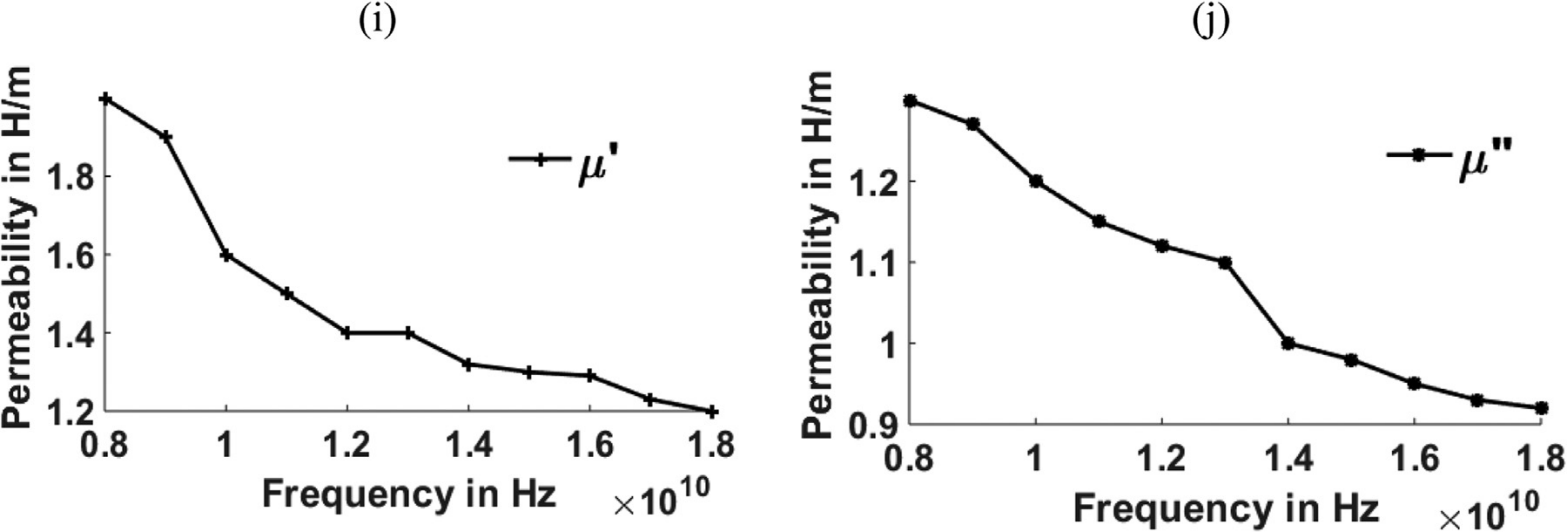
(i), (j)Relation between Permeability to frequency for Carbonyl iron.

**Fig. 8:**
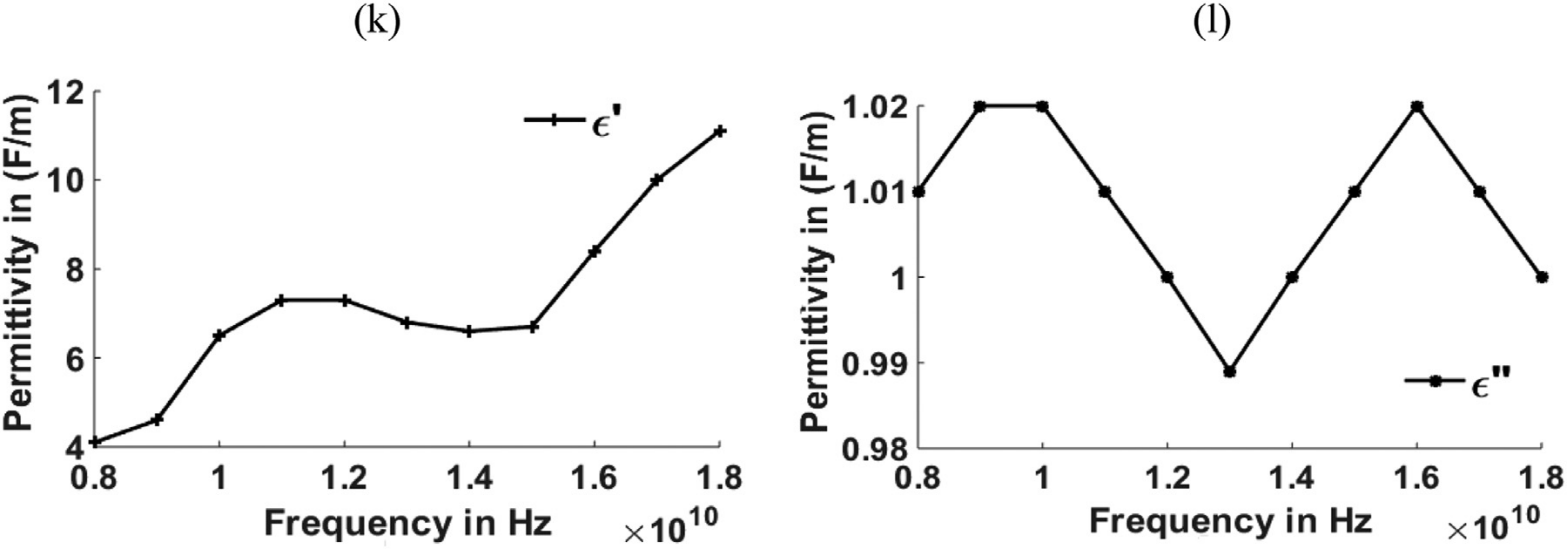
(k), (l) Relation between Permittivity to frequency for Carbonyl iron.

Fig. 3, Fig. 4, Fig. 5, Fig. 6, Fig. 7, Fig. 8 show the permeability and permittivity values of the micro absorbing material of MNZC ferrite rubber composite, Ca-NiTi hexaferrite composites, and Carbonyl iron as measured by a vector network analyzer employing reflection and transmission of transmitted waves.

The material's permittivity and permeability directly affect how effective the shielding is. The permittivity of the material can be improved by increasing the porosity, which can be increased by reinforcing the SiC material in to composite material [28]. High permeability materials exhibit anti spin to waves passing through them, which causes electromagnetic waves passing through them to be absorbed. Having strong permeability and permittivity, the micro-absorbing materials used here have good absorbing capabilities for electromagnetic waves. Al_2_O_3_ material can be reinforced for improving the permeability of the material [29,30]. According to the graphs above, carbonyl iron has a permeability of 2H/m, and MNZC ferrite rubber composite has a high permeability of 3.2H/m. It exhibits a solid anti spin where an electromagnetic wave travels and absorbs the most signal. Electromagnetic waves are reflected when a substance with a lower permittivity mismatches two neighboring free space media. The low permittivity of carbonyl iron at lower frequencies leads to more reflections.

## Results of shielding effectiveness

The maximum and minimum shielding effectiveness values at different thicknesses are compared and shown in Table 1 to Table 3.

**Table 1:** Maximum and minimum shielding effectiveness of 1 mm absorber and 1 mm MMC.

S.No	Material Type	Maximum Shielding effectiveness (dB)	Minimum Shielding effectiveness(dB)
1	1mm-MNZC ferrite rubber composite; 1mm-Al6061	41.36	31.17
2	1mm-Ca-NiTi exaferrite composites; 1mm-Al6061	41.31	38.81
3	1mm-Carbonyl iron; 1mm-Al6061	44.49	33.76

**Table 2:** Maximum and minimum shielding effectiveness of 2 mm absorber and 1 mm MMC.

S.No	Material Type	Maximum Shielding effectiveness (dB)	Minimum Shielding effectiveness(dB)
1	2mm-MNZC ferrite rubber composite; 1mm-Al6061	45.65	35.22
2	2mm-Ca-NiTi exaferrite composites; 1mm-Al6061	45.56	43.3
3	2mm-Carbonyl iron; 1mm-Al6061	49.69	38.6

**Table 3:** Maximum and minimum shielding effectiveness of 3 mm absorber and 1 mm MMC.

S.No	Micro absorbing Material Type	Maximum Shielding effectiveness (dB)	Minimum Shielding effectiveness(dB)
1	1mm-MNZC ferrite rubber composite; 1mm-Al6061	49.95	38.13
2	1mm-Ca-NiTi exaferrite composites; 1mm-Al6061	49.91	46.89
3	1mm-Carbonyl iron; 1mm-Al6061	53.75	41.78

The skin depth of the material reduces as the conductivity rises. The skin depth increases as the frequency of the EM wave increases. The results show that as the frequency increases, the shielding effectiveness decreases. The anti-spin interaction of atoms with EM waves rises as permeability increases, skin depth lowers, and shielding effectiveness improves.

**Fig. 9:**
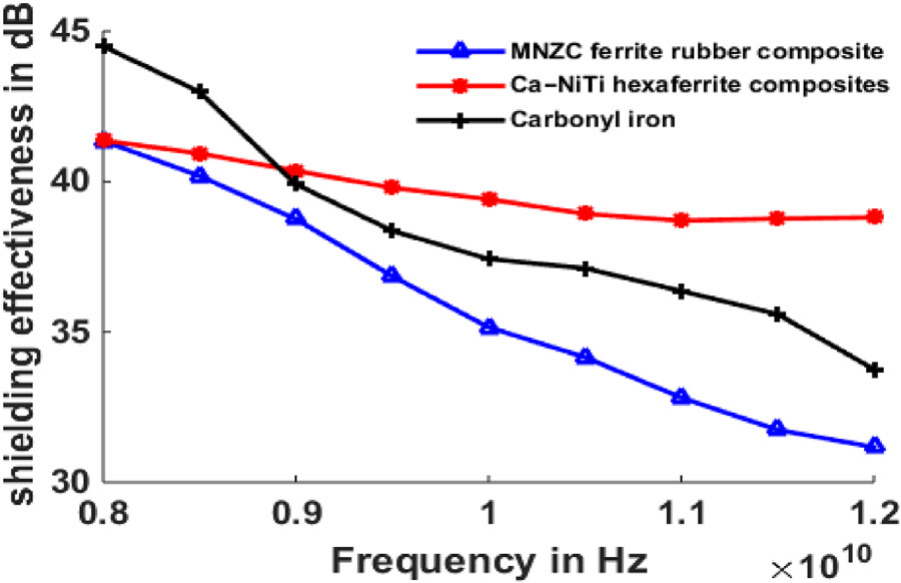
Shielding effectiveness of Al6061 and Micro absorbing material at 1 mm both.

**Fig. 10:**
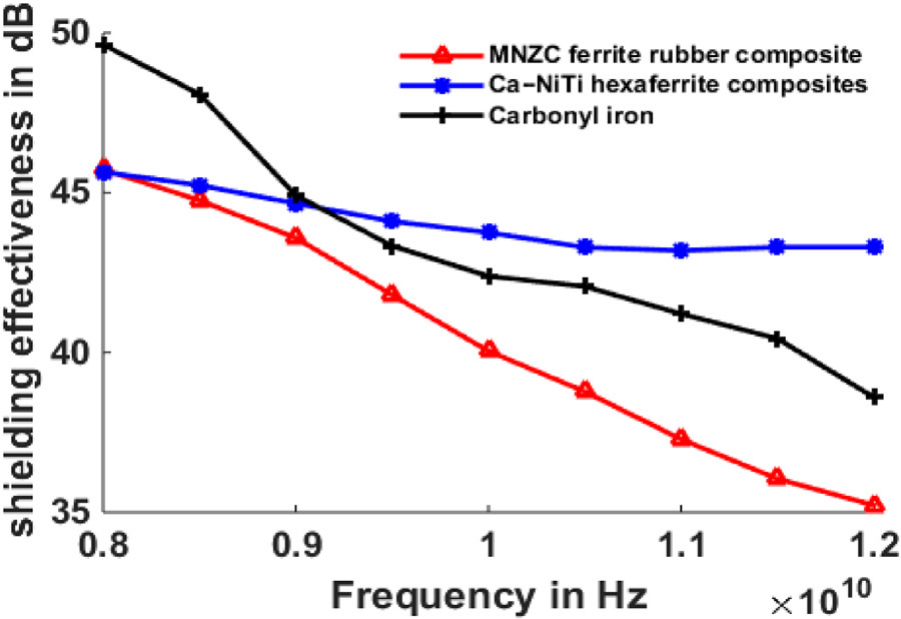
Shielding effectiveness of Al6061 1 mm and Micro absorbing material at 2 mm.

**Fig. 11:**
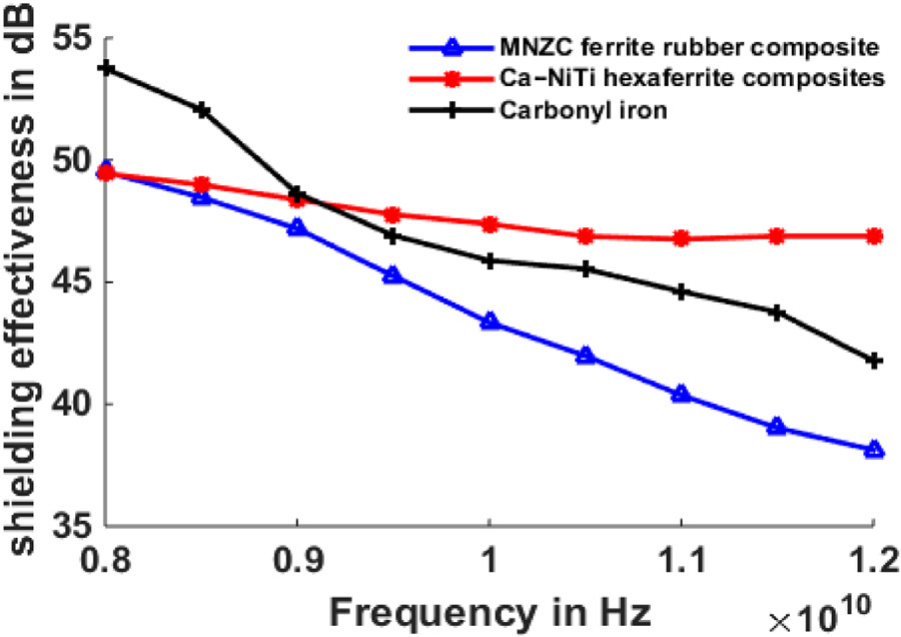
Shielding effectiveness of Al6061 1 mm and Micro absorbing material at 3 mm.

Fig. 9 depicts the shielding effectiveness of a Micro absorbing material supported by an Al6061 composite at 1 mm for normal incidence of electromagnetic waves in the X-band. Carbonyl iron was chosen as the absorbing material, with maximum shielding of 44.1 dB and lowest shielding of 33 dB at 8Ghz and 12Ghz, respectively. Maximums of 41 dB, 40 dB, and minimums of 31 dB and 39 dB were recorded for MNZC ferrite rubber composite, Ca-NiTi hexaferrite composites. The absorbing feature of the absorbing material is determined by its permeability; the magnitude of the permeability of carbonyl is more than that of MNZC ferrite rubber composites and Ca-NiTi hexaferrite composites, resulting in a more considerable shielding effectiveness value. Fig. 10 depicts a two-layer laminate of micro absorbent material supported by Al6061 with 2 mm and 1 mm thicknesses. In this case, the most incredible shielding efficiency was 48 dB for carbonyl iron and 46 dB for both. Fig. 11 depicts a two-layer laminate of micro absorbent material supported by Al6061 with thicknesses of 3 mm and 1 mm. The most excellent shielding performance of 54 dB for carbonyl iron and 49 dB for MNZC ferrite rubber composite, Ca-NiTi hexaferrite composites, was found here. Due to its absorption and reflection characteristics at lower frequencies, carbonyl iron provides the best shielding. Ca-NiTi hexaferrite composites have a maximum shielding of 46.89 dB at higher frequencies.

At 3 mm of micro absorbing and 1 mm of Al6061 material thickness, a two-layer laminate of micro absorbing material supported by Al6061 composite gives a maximum shielding of 54 dB. The laminate's weight is deficient due to the low densities of both absorbent materials and Al6061. The method was carried out for 2 mm and 1 mm thicknesses of micro absorbent material, yielding shielding values of 45.65 dB and 41.36 dB, respectively. The maximum of the propagating wave is absorbed during forwarding transmission, with the remainder absorbed following an incident on Al6061 sheet and reflected. At higher frequencies, Ca-NiTi hexaferrite and Al6061 composites demonstrate superior shielding characteristics, while Carbonyl iron and Al6061 laminate at lower frequencies. Greater than 30 dB shielding laminate can be employed for aerospace and military applications. Even Al6061 material has high hardness and tensile strength, which is an added benefit for hiring laminate in aircraft applications regularly.(Table 4)

**Table 4:** List of symbols.

Symbol	Description	Symbol	Description
$\eta_{O}$	Free space intrinsic impedance	$\mu_{M}$	Permeability of metal
$\eta_{A}$	Absorbing material intrinsic impedance	$\sigma_{M}$	Metal conductivity
$\;\eta_{M}$	Metal intrinsic impedance	$t_{A}$	Absorber Thickness
$\gamma_{A}$	Propagation constant of absorber	$t_{C}$	Conductor Thickness
$\gamma_{C}$	Propagation constant of conductor	f	Frequency of operation

## Declaration of Competing Interest

The authors declare that they have no known competing financial interests or personal relationships that could have appeared to influence the work reported in this paper.

## Data Availability

Data will be made available on request.
